# Psychometric assessment of the Persian adaptation of the attitudes toward seeking professional psychological help scale-short form

**DOI:** 10.1186/s12888-023-05388-2

**Published:** 2024-01-26

**Authors:** Abouzar Nazari, Gholamreza Garmaroudi, Abbas Rahimi Foroushani, Azadeh Askari

**Affiliations:** 1 Department of Health Education and Promotion, Faculty of Health, Tehran University of Medical Sciences, Tehran, Iran; 2 Department of Health Education and Promotion, School of Health, Tehran University of Medical Sciences, Tehran, Iran; 3 Department of Epidemiology and Biostatistics, School of Health, Tehran University of Medical Sciences, Tehran, Iran; 4 Department of Psychology, Faculty of Psychology and Educational Sciences, Shahid Beheshti University, Tehran, Iran

**Keywords:** Psychometrics, Validity, Reliability, Confirmatory factor analysis, Mental health services, Attitudes toward seeking Professional Psychological Help Scale

## Abstract

**Background and purpose:**

Mental health disorders are a growing concern worldwide, with a significant impact on public health. Understanding attitudes toward seeking professional psychological help is essential in addressing these issues. In the Iranian context, there is a need for a reliable tool to measure these attitudes. This study aims to assess the validity and reliability of the Persian Adaptation of the Attitudes Toward Seeking Professional Psychological Help Scale-Short Form (ATSPPH-SF).

**Materials and methods:**

A cross-sectional study was conducted in May 2023, utilizing a convenience sampling method with 1050 participants aged 10 to 65 years in Iran.The ATSPPH-SF questionnaire, consisting of 10 items and 2 subscales, was employed. The questionnaire underwent translation and cultural adaptation, and its validity was assessed through qualitative face and content validities. Confirmatory factor analysis (CFA) was used to evaluate construct validity. Reliability was assessed using McDonald’s omega coefficient and Cronbach’s alpha coefficient. Data collection was conducted through an online survey.

**Results:**

The CFA results indicated a two-factor structure for the ATSPPH-SF, with one factor representing openness to seeking treatment for emotional problems and the other factor reflecting the value and need for seeking treatment. The model demonstrated acceptable fit indices. Both McDonald’s omega coefficient and Cronbach’s alpha coefficient suggested good internal consistency for the scale. The mean total score for the ATSPPH-SF was 21.37 (SD = 5.52), indicating the reliability and validity of the scale for the Iranian population.

**Conclusion:**

This study confirms the suitability of the short-form ATSPPH-SF with 10 items and 2 subscales as a valid and reliable tool for assessing attitudes toward seeking professional psychological help in the Iranian population. With no prior appropriate instrument available, this scale fills a crucial gap. It can be employed to measure attitudes among various demographic groups, aiding in the design of targeted interventions to enhance mental health literacy and reduce the stigma associated with seeking professional psychological help in Iran.

## Introduction

Mental health is a crucial component of overall well-being, impacting individuals, families, and communities across the globe [1, 2]. As societies evolve, understanding and addressing attitudes toward seeking professional psychological help becomes increasingly vital [3–5]. This study is motivated by the importance of exploring such attitudes within the Iranian population, as they navigate the complex interplay between cultural influences, personal beliefs, and societal factors in their approach to mental health support [6, 7].

### Global context of attitudes toward seeking professional psychological help

The prevalence of mental health disorders and the associated burden they impose are well-documented on a global scale. Worldwide, mental health issues account for a substantial proportion of the global disease burden and contribute to reduced quality of life, increased mortality, and decreased overall productivity. It is evident that the widespread impact of mental health disorders necessitates a comprehensive approach, including improving attitudes toward seeking professional psychological help [8–10].

### Specific challenges in the Iranian context

In Iran, as in many other countries, mental health issues represent a significant public health concern. Despite the increasing recognition of these issues, attitudes toward seeking professional psychological help in Iran remain an area requiring exploration and understanding. Reviewers have previously noted that limited studies have addressed this specific challenge, making it essential to delve into the attitudes and perceptions of Iranians regarding professional mental health support [11, 12].

### Rationale for the study

To address the aforementioned gaps in knowledge, this study focuses on the adaptation and psychometric evaluation of the Attitudes Toward Seeking Professional Psychological Help Scale-Short Form (ATSPPH-SF) within the Iranian context. The ATSPPH-SF is a widely-used instrument for assessing attitudes toward seeking professional psychological help, providing insights into individuals’ willingness and openness to obtaining mental health support. Given the cultural and contextual variations observed in attitudes toward mental health, this research aims to validate a tool that aligns with the Iranian cultural landscape and is tailored to the unique characteristics of the Iranian population. [13].

### Adaptation of the ATSPPH-SF

An essential step in ensuring that a measurement tool is fit for use in a new cultural context is its adaptation and validation. The successful adaptation of the ATSPPH-SF for use in Iran hinges on rigorous translation processes, cultural relevance, and rigorous psychometric evaluation. Through this process, we aim to provide a reliable and valid tool that can assist in assessing and improving attitudes toward seeking professional psychological help among Iranians. [14–16].

### Bridging the knowledge gap

This study seeks to bridge the knowledge gap by systematically assessing the psychometric properties of the adapted ATSPPH-SF within the Iranian general population. The investigation delves into aspects of face validity, content validity, construct validity, and reliability to ensure that the adapted tool effectively measures attitudes relevant to the Iranian context. By doing so, this research endeavors to provide a valid and reliable instrument for screening, assessment, and intervention purposes in Iranian mental health settings.

In summary, this study strives to address the unique challenges faced by the Iranian population concerning attitudes toward seeking professional psychological help. By adapting and validating the ATSPPH-SF in this context, we aim to contribute to a growing body of knowledge, potentially improving mental health services and attitudes in Iran, and offering a blueprint for similar research in other culturally diverse populations.

## Materials and methods

### Study design and participants

This This cross-sectional psychometric study aimed to assess the validity and reliability of the Iranian version of the Attitudes Toward Seeking Professional Psychological Help Scale-Short Form (ATSPPH-SF) within the general population. The study was conducted in May 2023, utilizing a convenience sampling method. The sample consisted of 1050 individuals who met the inclusion criteria, which required them to be between the ages of 10 and 65 years and to have provided informed consent to participate in the study.

### Sample size calculation

The sample size of 1050 participants in this study was determined based on convenience sampling, which does not rely on a specific software, formula, or citation to an article. Convenience sampling was chosen as a practical method for this exploratory study, and the sample size was considered sufficient for assessing the psychometric properties of the ATSPPH-SF within the scope of this research.

### Data collection

We employed an online survey hosted on Porsline’s website to gather data for this study. Porsline is a web-based platform that facilitates the creation, distribution, data collection, and analysis of surveys for researchers. Additionally, Porsline offers features that enable researchers to obtain informed consent from participants in a secure and ethical manner. We created and hosted our questionnaire on Porsline, making it accessible for participants to complete online. To enhance participation from the general population, we shared the survey link across multiple social media platforms.

### Instruments

The data collection instruments included:

1. Demographic Questionnaire: This questionnaire encompassed items related to participants’ gender, age, level of education, father’s education level, mother’s education level, economic situation, number of family members, and other relevant demographic information.
2. Attitudes Toward Seeking Professional Psychological Help-Short Form (ATSPPH-SF): The ATSPPH-SF is a concise questionnaire with 10 items designed to assess individuals’ attitudes toward seeking professional help for mental health issues. Each item is rated on a 4-point scale, ranging from 0 to 3. A higher total score indicates a more positive attitude and reduced stigma toward mental health issues. The ATSPPH-SF consists of two aspects: openness to seeking professional help for emotional problems (items 1, 3, 5, 6, 7) and the value and need for professional help (items 2, 4, 8, 9, 10). The total score on the ATSPPH-SF can range from 10 to 40, with higher scores indicating a more positive attitude toward seeking professional psychological help. For interpretation, scores above 20 are indicative of a positive attitude, while scores below 20 suggest a less positive attitude toward seeking professional psychological help. [13, 17].

Table 1 is designed to display the Promax rotated maximum likelihood factor loadings for the Attitudes Toward Seeking Professional Psychological Help Scale-Short Form (ATSPPH-SF). This table provides insights into the factor structure of the ATSPPH-SF questionnaire and how individual items load onto the identified factors. The data presented in Table 1 were derived from the results of the Confirmatory Factor Analysis (CFA) conducted during the study, and the factor loadings were based on the responses from the study participants [13].

**Table 1:** Promax rotated maximum likelihood factor loadings for the Attitudes Toward Seeking Professional Psychological Help Scale-Short Form (ATSPPH-SF).

ATSPPH-SF	Factor#1: Openness	Factor#2: Value and Need
Item #1: Would obtain professional help if having a mental breakdown	0.74	− 0.02
Item #2: Talking about psychological problems is a poor way to solve emotional problems	0.19	0.54
Item #3: Would find relief in psychotherapy if in emotional crisis	0.72	0.08
Item #4: A person coping without professional help is admirable	− 0.13	0.50
Item #5: Would obtain psychological help if upset for a long time	0.73	− 0.07
Item #6: Might want counseling in the future	0.47	0.06
Item #7: A person with an emotional problem is likely to solve it with professional help	0.43	0.05
Item #8: Psychotherapy would not have value for me	0.01	0.51
Item #9: A person should work out his/her problems without counseling	0.01	0.76
Item #10: Emotional problems resolve by themselves	− 0.02	0.62

### Translation and cultural adaptation

A rigorous process was employed for the translation and cultural adaptation of the ATSPPH-SF. The forward-backward method was utilized [18]. Initially, two independent experts performed separate translations of the original English version of the questionnaire into Persian. Subsequently, the translated versions were reconciled, resulting in a single Persian version of the questionnaire. An English language expert, unfamiliar with the psychology-specific content, performed a back-translation into English, and the English back-translation was compared to the original English version. Finally, the English translation was re-translated into Persian by two psychology specialists proficient in the English language. The questionnaire’s validity and reliability were thoroughly assessed during this process.

### Validation

The questionnaire utilized in this study, the Attitudes Toward Seeking Professional Psychological Help Scale-Short Form (ATSPPH-SF), is a standardized instrument with established reliability and validity [19]. The validation process for this questionnaire involved an assessment of its qualitative face and content validity, which were crucial for ensuring its cultural appropriateness and linguistic precision while retaining the original scale’s core structure.

Qualitative Face Validity: To evaluate qualitative face validity, the questionnaire underwent review by a panel of 10 individuals who represented the target population. This panel assessed the questionnaire items in terms of ambiguity, relevance, suitability, and question difficulty. Their feedback was instrumental in identifying areas for improvement and enhancing the clarity and cultural relevance of the questionnaire. Subsequent modifications were implemented based on their valuable insights.

Qualitative Content Validity: The assessment of qualitative content validity was conducted by submitting the questionnaire to 13 specialists in the fields of public health and health education. These experts conducted a comprehensive evaluation, taking into account various attributes, including grammar, word choice, item importance, the time required to respond to each question, item placement, and other relevant factors. Their expertise contributed significantly to refining the questionnaire’s content and ensuring it met the highest standards of quality.

This iterative process of review and feedback, involving both members of the target population and subject matter experts, allowed us to enhance the questionnaire’s quality. It ensured that the questionnaire was culturally appropriate, linguistically precise, and retained its core meaning and structure from the original scale.

The rigorous validation process underscores the questionnaire’s suitability for our study, taking into account the cultural diversity of the Iranian population. It also demonstrates our commitment to obtaining accurate and reliable data for our research.

### Confirmatory factor analysis (CFA)

The study employed CFA to evaluate construct validity. Prior to CFA, the data were examined for outliers using Mahalanobis statistics. Normality was assessed using skewness and kurtosis. CFA was conducted using AMOS version 24 software. Items with weak internal consistency were excluded from the questionnaire to obtain an acceptable model. Items with factor loadings lower than 0.3 were removed to achieve an acceptable final model [20].

Several fit indices were used to assess the model, including the chi-square ratio to the degree of freedom (χ²/df), root mean square error of approximation (RMSEA), goodness of fit index (GFI), parsimonious normed fit index (PNFI), parsimony comparative fit index (PCFI), incremental fit index (IFI), comparative fit index (CFI), and parsimonious normed fit index (PNFI). The model was considered acceptable if it met the criteria of χ²/df < 4, RMSEA ≤ 0.08, PCFI, PNFI > 0.5, TLI > 0.95, and other indices of IFI and CFI > 0.9. [21–24].

### Reliability assessment

The internal consistency of the ATSPPH-SF and its individual attributes was assessed using McDonald’s omega coefficient and Cronbach’s alpha coefficient. McDonald’s omega coefficient was calculated using SPSS version 24 software, as it provides a more precise estimate of internal consistency than Cronbach’s alpha coefficient [25]. We considered reliability coefficients above 0.70 as acceptable, aligning with the recognized standards for developing a new measure [26]. Moreover, the minimum criterion for the internal reliability of the questionnaire was set at a Cronbach’s alpha coefficient of 0.60 [27]. It’s important to note that lower values of McDonald’s omega coefficient and Cronbach’s alpha coefficient may be observed for attributes with a smaller number of items.

This comprehensive reliability assessment underscores the internal consistency and stability of the ATSPPH-SF and its individual attributes. It provides confidence in the reliability of this instrument, as the majority of attributes met or exceeded the acceptable threshold, ensuring that it is well-suited for assessing attitudes toward seeking professional psychological help in our study.

**Fig. 1:**
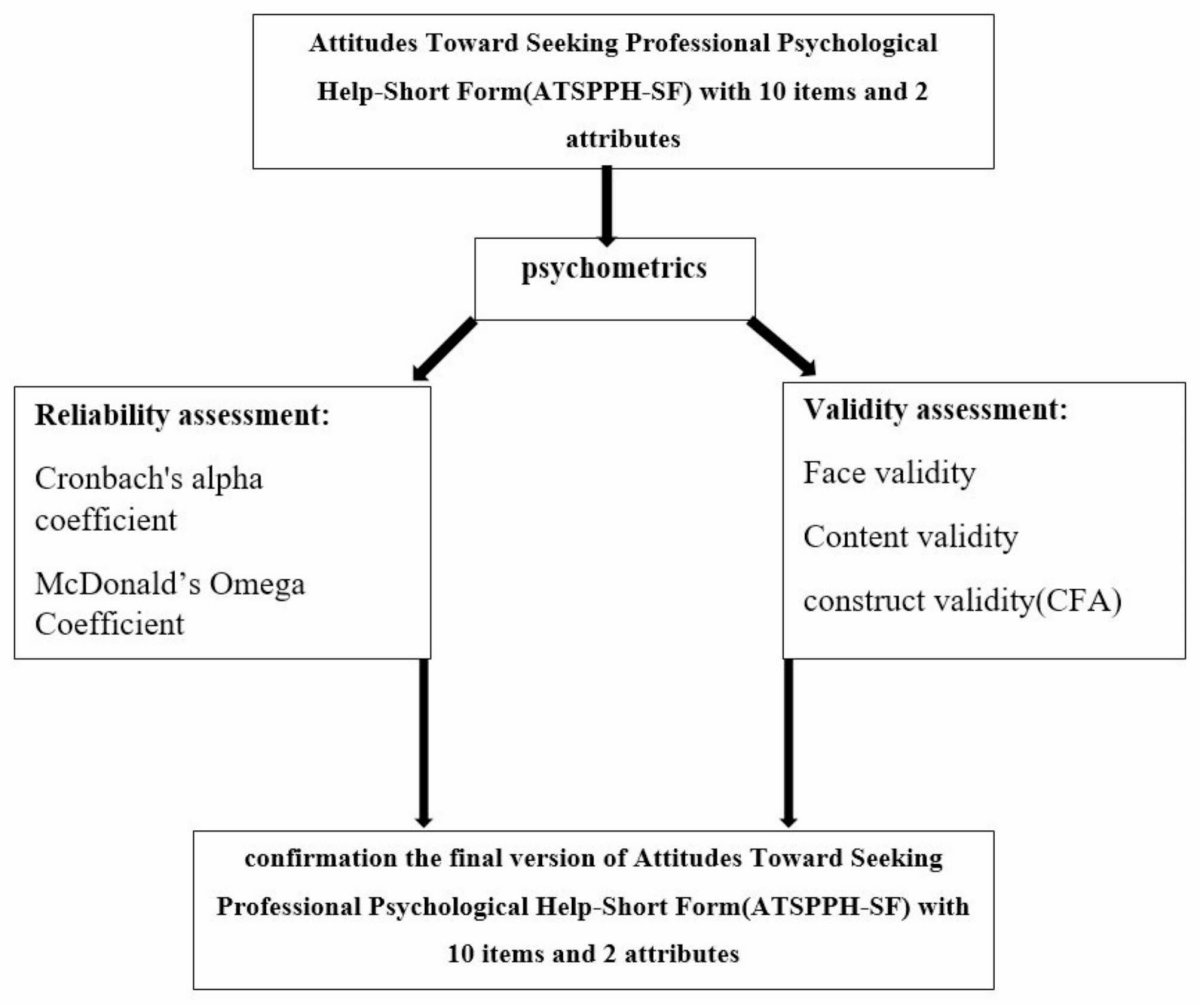
A summary of the modifying of ATSPPH-SF

A summary of the modifications made to the ATSPPH-SF is presented in Fig. 1.

## Results

### Demographic characteristics

A summary of the demographic characteristics of the study participants is presented in Table 2. Of the 1050 participants, 57.4% were female, with 42.6% being male. The participants’ age had an average (standard deviation) of 29.87 (7.98) years, with the youngest participant being 10 years old and the oldest being 65 years old. In terms of education, 29.4% had a diploma or lower qualifications, 49.7% held an associate or bachelor’s degree, and 20.9% had a master’s or Ph.D. degree. Family members’ education levels varied, with 73.9% of fathers having a diploma or lower qualifications, 20.0% with an associate or bachelor’s degree, and 6.1% with a master’s or Ph.D. degree. For mothers, 82.1% had a diploma or lower qualifications, 14.7% held an associate or bachelor’s degree, and 3.2% had a master’s or Ph.D. degree. Economic status distribution showed 13.7% of participants had a weak economic status, 60.3% had an average economic status, and 26% had a good economic status. The mean (standard deviation) age of the participants was 29.87 (7.98) years, and the average number of family members was 4.75 (1.78).

**Table 2:** Frequency distribution of demographic characteristics (n = 1050)

Variables		N	%
Gender	Female	606	57.4
	Male	449	42.6
Level of education	Diploma / less	310	29.4
	Associate /bachelor’s degree	524	49.7
	Masters / PhD	220	20.9
Father’s level of education	Diploma / less	780	73.9
	Associate / bachelor’s degree	211	20.0
	Masters / PhD	64	6.1
Mother’s level of education	Diploma / less	866	82.1
	Associate / bachelor’s degree	155	14.7
	Masters / PhD	34	3.2
Father’s job	Employee (public or private)	147	13.9
	Manual worker	114	10.8
	Retired	409	38.8
	Unemployed	51	4.8
	Other	334	31.7
Mother’s job	Employee (public or private)	73	6.9
	Manual worker	13	1.2
	Retired	87	8.2
	Housewife	802	76.0
	Other	80	7.6
Economic status of the family	poor	145	13.7
	average	636	60.3
	Good	274	26

### Qualitative validity assessment (face and content validity)

No question was deleted during the translation and cultural adaptation processes because the subject’s statements in the original questionnaire were similar to the culture of the Iranian population. During the processes of face and content validities’ assessment, the questionnaire was given to 13 specialists (from the fields of psychology, and health education and promotion). As a result, four items were corrected based on their feedback. The corrections included changing the wording of some items to make them more clear and understandable (For example, we translated professional help to expert help to create the target meaning.), and modifying the grammatical structure of some sentences to make them more consistent with the Persian language. (Fig. 1).

### Confirmatory factor analysis

The ATSPPH-SF’s factor structure should have one or two factors in theory. Its items were related to one underlying factor in the study that developed the short form (Fischer and Farina, 1995), which supports a one-factor solution. However, the items were only taken from two of the original scale’s four factors: Recognition of Need for Psychotherapeutic Help, and Confidence in Mental Health Practitioner (Fischer and Turner, 1970), which supports a two-factor structure.

**Fig. 2:**
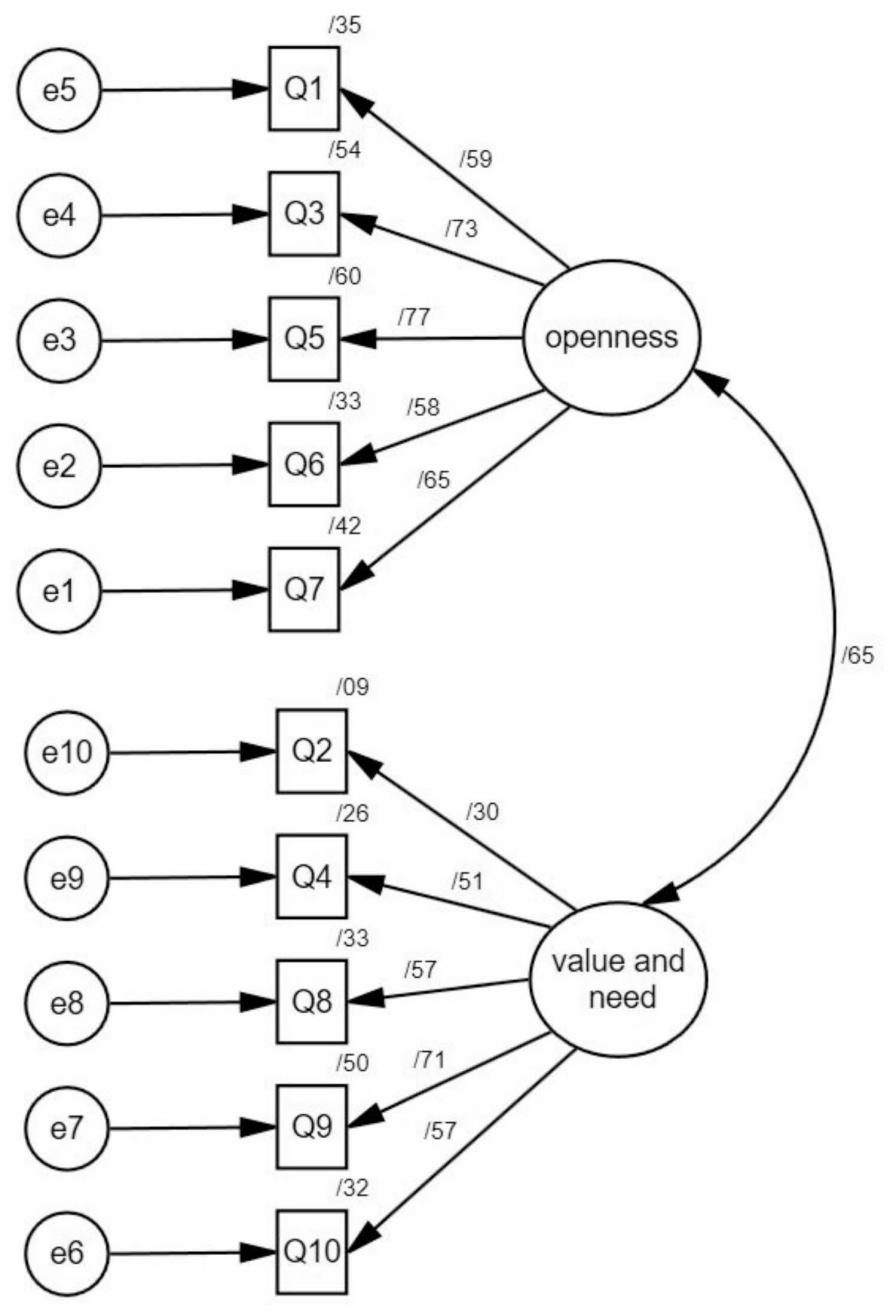
Standardized parameter estimates for the factor structure of the ATSPPH-SF

We tested these one- and two-factor structures with maximum likelihood confirmatory factor analyses (CFA). The one-factor structure, based on previous research, demonstrated poor factor loadings (with five items having factor loadings less than 0.4) and unsatisfactory fit indices (χ²/df = 252.427/25, CFI = 0.879, TLI = 0.884, RMSEA = 0.055, SRMR = 0.047). In contrast, the two-factor structure, incorporating items from the original scales “Recognition of Need for Psychotherapeutic Help” and “Confidence in Mental Health Practitioner,“ revealed an acceptable fit with a good range of fit indices (χ²/df = 3.542, RMSEA = 0.049, PCFI = 0.729, PNFI = 0.720, CFI = 0.965, TLI = 0.954, IFI = 0.965) (see Table 3 for details). In the CFA stage, one of the items (question 2) had a factor loading of less than 0.4, but we did not remove it because of the suitability of other fit indices. The factor loading value of each question is shown in Fig. 2.

**Table 3:** The model fit indicators of ATSPPH-SF

Goodness of fit indices	Confirmatory factor analysis	Acceptable value
X^2^/df *p*-value	3.542 0.000	< 4 *p* < 0.05
CFI TLI IFI RMSEA PNFI PCFI	0.965 0.954 0.965 0.049 0.720 0.729	> 0.95 > 0.95 > 0.9 < 0.06 > 0.5 > 0.5

The final version of the ATSPPH-SF included a total of 10 items, including Openness to Seeking Treatment for Emotional Problems (5 items) and Value and Need in Seeking Treatment (5 items).

### Reliability assessment

Reliability assessments were conducted using both McDonald’s omega coefficient and Cronbach’s alpha coefficient for the entire ATSPPH-SF and its attributes, which include openness to seeking treatment for emotional problems and value and need in seeking treatment. The reliability results indicate that the entire scale showed good internal consistency (McDonald’s omega = 0.785, Cronbach’s alpha = 0.789). Additionally, the attributes demonstrated good reliability: openness scale (McDonald’s omega = 0.803, Cronbach’s alpha = 0.795) and value and need scale (McDonald’s omega = 0.659, Cronbach’s alpha = 0.656). The test-retest reliability, conducted over a two-week period, was 0.855 for the entire scale, 0.741 for the openness scale and 0.787 for the value and need scale (see Table 4).

These results confirm that the ATSPPH-SF, following translation and adaptation, exhibits good psychometric properties, making it a reliable and valid tool for assessing Attitudes Toward Seeking Professional Psychological Help Scale in the Iranian context.

**Table 4:** Descriptive statistics of the ATSPPH-SF and its attributes

Attributes	Item	Mean	SD	Cronbach’s alpha	McDonald’s omega
Openness to Seeking Treatment for Emotional Problems Value and Need in Seeking Treatment	5 5	11.52 9.85	3.14 3.31	0.795 0.656	0.803 0.659
The final modified version of ATSPPH-SF (All attributes)	10	21.37	5.52	0.789	0.785

## Discussion

The primary objective of this study was to assess the psychometric properties of the ATSPPH-SF among the Iranian general population and to provide a valid and reliable tool for measuring and improving attitudes toward seeking professional psychological help in this population. This validated scale offers promising prospects for screening, assessment, and intervention purposes in mental health settings within Iran.

### Interpretation of factor structure

The factor structure revealed a two-factor model, consistent with the findings of Elhai et al. [17]. These factors represent “Perceived Stigma” and “Perceived Psychological Openness.“ In the Iranian context, “Perceived Stigma” may underscore the influence of cultural factors such as stigma, shame, and self-reliance. The lower scores on “Perceived Psychological Openness” suggest a potential lack of recognition of the value and need for professional psychological help, reflecting cultural nuances. This finding highlights the importance of addressing these cultural barriers in promoting positive attitudes toward seeking help in Iran.

### Perceived stigma

In the context of the two-factor structure, the “Perceived Stigma” factor signifies that individuals in Iran may harbor concerns about social repercussions and negative judgment when considering professional psychological help. These concerns might be rooted in societal attitudes, which could perpetuate stereotypes about mental health issues. Addressing this aspect of stigma is crucial, as it can deter individuals from seeking help when needed. Public awareness campaigns, education, and open discussions about mental health can play a significant role in diminishing perceived stigma. [12, 28–30].

### Perceived psychological openness

The “Perceived Psychological Openness” factor encapsulates the extent to which individuals in Iran recognize the value and necessity of professional psychological help. A lower score on this factor suggests that there might be room for improving the acknowledgment of the positive impact that seeking professional psychological help can have on mental well-being. This calls for interventions aimed at elucidating the advantages of early intervention and destigmatizing mental health services. Encouraging open conversations within families, communities, and educational institutions can contribute to a more accepting and supportive atmosphere. [28, 29, 31].

### Cultural variations in attitudes

The differences in factor structures between our study and others [13, 32, 33], emphasize the significance of adapting and validating the scale within specific cultural contexts. The variations in attitudes toward seeking professional psychological help among different populations and settings further underline the need for culturally sensitive and tailored interventions.

These cultural variations may be driven by complex sociocultural factors unique to the Iranian context. Therefore, future research should delve into the specific cultural and contextual factors that influence attitudes toward mental health help-seeking in Iran. Qualitative studies, focus groups, and in-depth interviews could provide a richer understanding of the multifaceted cultural dynamics at play.

### Reliability and consistency

The reliability of the ATSPPH-SF was consistent with previous studies, demonstrating good internal consistency and stability over time. This aligns with findings from Elhai [17], Picco [32], and Fischer and Farina [13]. These results collectively indicate that the scale can provide consistent and accurate results across different situations and samples.

### Implications for mental health in Iran

The moderately positive attitude toward seeking professional psychological help indicates room for improvement, particularly in recognizing the value and need for such help. Cultural factors, including stigma, shame, and self-reliance, may contribute to these findings, reflecting the complex interplay between attitudes and cultural norms in Iran [30–32, 34].

### Addressing cultural barriers

The low score on the “Perceived Psychological Openness” factor underlines the importance of addressing cultural barriers to seeking professional psychological help. Stigma, a well-documented obstacle, is one of the main barriers to seeking and utilizing mental health services in Iran. People with mental health problems may face negative social reactions, discrimination, and isolation, all of which can deter them from seeking help [34, 35].

### Promoting awareness and access

Efforts to raise awareness, reduce stigma, and increase access to mental health services are essential. Strategies should encompass public education about the nature, causes, and treatments of mental disorders, the promotion of positive attitudes and behaviors toward people with mental health problems, and the provision of accessible, affordable, and culturally appropriate mental health services. Additionally, engaging family, community, and religious leaders in the prevention and intervention of mental health problems can be instrumental in challenging prevailing cultural norms and fostering a supportive environment for those seeking help. [36].

### Future directions

To continue advancing our understanding of attitudes toward seeking professional psychological help in Iran, future research should explore the predictive validity of the ATSPPH-SF. This involves investigating how well the scale can predict actual help-seeking behavior and outcomes among individuals with mental health concerns. This could provide insights into the real-world impact of attitudes on help-seeking behavior.

### Incorporating qualitative research

A deeper exploration of individual experiences and cultural nuances can be achieved through qualitative research. Conducting interviews and focus groups with diverse segments of the Iranian population can yield valuable qualitative data that complements the quantitative findings. Such studies can provide a more comprehensive understanding of the factors that influence attitudes toward seeking professional psychological help [37, 38].

### Subpopulation analysis

Future studies should examine the validity and reliability of the ATSPPH-SF in various subgroups of the Iranian population, such as ethnic minorities, rural residents, or people with specific mental disorders. These analyses can help uncover variations in attitudes and needs among different segments of the population.

### Limitations

The study had some limitations that warrant attention. One of them was the use of social media to distribute the questionnaire, which might have excluded people who were not active or present on these platforms. This could have reduced the inclusivity of the study by leaving out a part of the population. Another limitation was the dependence on self-rating scales to measure different aspects, such as depressive symptoms, stigma related to depression, and help-seeking attitudes. These self-report measures might have introduced response bias, as participants could have answered according to social expectations or norms, which might have affected the validity of our findings. Despite these limitations, the study’s weaknesses should also be recognized. The sample size, though adequate for a preliminary assessment, might not reflect the diversity of the Iranian population. Our convenience sampling method might have caused selection bias, which further limited the applicability of our results. Moreover, the cross-sectional study design prevented us from establishing causal relationships. We suggest future research to use more diverse and comprehensive sampling methods to enhance the external validity of the study and enable the inference of causality.

## Conclusion

In conclusion, this study has validated the Persian version of the ATSPPH-SF, comprising 10 items and 2 factors, for assessing attitudes toward seeking professional psychological help among the Iranian general population. This achievement marks a significant step toward promoting mental health awareness, reducing stigma, and enhancing access to mental health services in Iran. The results emphasize the need for comprehensive efforts to improve attitudes toward seeking professional psychological help in the country. Strategies encompass public education, the promotion of positive attitudes and behaviors, accessible mental health services, and engagement with community and religious leaders. These initiatives hold the potential to enhance mental health and well-being across the Iranian population, ultimately improving the lives of individuals and the broader community.

## Data Availability

The data sets used and/or analyzed during the current study were available from the corresponding author on reasonable request.

## Abbreviations

ATSPPH: Attitudes Towards Seeking Professional Psychological Help
ATSPPH-SF: Attitudes Towards Seeking Professional Psychological Help-Short Form
CFA: confirmatory factor analysis
